# Clinical role of intraperitoneal chemotherapy in patients with pancreatic ductal adenocarcinoma concomitant with occult peritoneal dissemination: A multicenter retrospective study

**DOI:** 10.1002/ags3.70001

**Published:** 2025-03-04

**Authors:** Tomohisa Yamamoto, Toshio Shimokawa, Masamichi Hayashi, Masamichi Mizuma, Katsuhisa Hirano, Atsushi Oba, Toshimichi Asano, Hideyo Miyato, Makoto Yoshida, Ippei Matsumoto, Yasunari Kawabata, Katsunori Sakamoto, Fuyuhiko Motoi, Shigeto Ishii, Yuki Homma, Hiromitsu Maehira, Yutaro Matsunaga, Tetsuya Ikemoto, Masafumi Nakamura, Yuko Mataki, Tsuyoshi Notake, Keiichi Akahoshi, Hideki Takami, So Yamaki, Daisuke Hashimoto, Yasutoshi Kimura, Satoshi Hirano, Yosuke Inoue, Tsutomu Fujii, Michiaki Unno, Yasuhiro Kodera, Joji Kitayama, Sohei Satoi, the Study Group of Pancreatic Ductal Adenocarcinoma with Peritoneal Dissemination

**Affiliations:** ^1^ Department of Pancreatobiliary Surgery Kansai Medical University Osaka Japan; ^2^ Clinical Study Support Center, Wakayama Medical University School of Medicine Wakayama Japan; ^3^ Department of Gastroenterological Surgery Nagoya University Graduate School of Medicine Nagoya Japan; ^4^ Department of Surgery Tohoku University Graduate School of Medicine Sendai Japan; ^5^ Department of Surgery and Science, Faculty of Medicine, Academic Assembly University of Toyama Toyama Japan; ^6^ Division of Hepatobiliary and Pancreatic Surgery Cancer Institute Hospital, Japanese Foundation for Cancer Research Tokyo Japan; ^7^ Department of Gastroenterological Surgery II, Faculty of Medicine Hokkaido University Sapporo Japan; ^8^ Department of Gastrointestinal Surgery Jichi Medical University Shimotsuke Japan; ^9^ Department of Medical Oncology Sapporo Medical University School of Medicine Sapporo Japan; ^10^ Department of Surgery Kindai University Faculty of Medicine Osaka Japan; ^11^ Department of Digestive and General Surgery Shimane University Faculty of Medicine Izumo Japan; ^12^ Department of Hepato‐Biliary‐Pancreatic and Breast Surgery Ehime University Graduate School of Medicine Ehime Japan; ^13^ Department of Surgery Yamagata University Graduate School of Medical Science Yamagata Japan; ^14^ Department of Gastroenterology, Graduate School of Medicine Juntendo University Tokyo Japan; ^15^ Department of Gastroenterological Surgery Yokohama City University Graduate School of Medicine Yokohama Japan; ^16^ Department of Surgery Shiga University of Medical Science Otsu Japan; ^17^ Department of Surgery, Institute of Gastroenterology Tokyo Women's Medical University Tokyo Japan; ^18^ Department of Surgery Tokushima University Tokushima Japan; ^19^ Department of Surgery and Oncology, Graduate School of Medical Sciences Kyushu University Fukuoka Japan; ^20^ Department of Digestive Surgery, Graduate School of Medicine Kagoshima University Kagoshima Japan; ^21^ Division of Gastroenterological, Hepato‐Biliary‐Pancreatic, Transplantation, and Pediatric Surgery, Department of Surgery Shinshu University School of Medicine Nagano Japan; ^22^ Department of Hepatobiliary and Pancreatic Surgery, Graduate School of Medicine Tokyo Medical and Dental University Tokyo Japan; ^23^ Department of Surgery, Surgical Oncology and Science Sapporo Medical University School of Medicine Sapporo Japan; ^24^ Division of Surgical Oncology University of Colorado Anschutz Medical Campus Aurora Colorado USA

**Keywords:** intraperitoneal chemotherapy, peritoneal dissemination

## Abstract

**Background:**

The effectiveness of intraperitoneal chemotherapy using paclitaxel (i.p.‐PTX) in pancreatic ductal adenocarcinoma (PDAC) patients with peritoneal dissemination remains elusive. The aim of this study is to investigate the clinical outcome of patients treated with i.p.‐PTX combined with systemic chemotherapy compared with current standard chemotherapy including gemcitabine plus nab‐paclitaxel and FOLFIRINOX.

**Methods:**

Data of patients with peritoneal dissemination was retrospectively collected and analyzed (i.p.‐PTX, *n* = 83; control, *n* = 86). Inverse probability of treatment‐weighted analyses (IPTW) was used to balance baseline characteristics between two groups. Survival curves were estimated using Kaplan–Meier method, and the differences were compared using the log‐rank test.

**Results:**

No significant differences were noted in overall survival (14.9 vs. 15.5 months, *p* = 0.481) and progression free survival (9.5 vs. 9.1 months, *p* = 0.267) between i.p.‐PTX and the control groups. Nevertheless, i.p.‐PTX (9.9 months) significantly prolonged the median progression‐free survival (PFS) time compared with the control (8.6 months), among the matched patients using IPTW (hazard ratio 0.666, *p* = 0.041). Moreover, subgroup analysis among the patients whose primary tumor were evaluated either as resectable or borderline resectable disease revealed significantly better overall survival in the i.p.‐PTX group compared with the control group (21.3 vs. 14.7 months, hazard ratio; 0.532, *p* = 0.033). Conversion surgery was more frequently performed in the i.p.‐PTX group than the control group (24% vs. 4%, *p* = 0.006).

**Conclusion:**

The i.p. PTX regimen prolonged PFS but not overall survival, and subgroup analysis suggested the possibility of survival benefit in patients with occult peritoneal dissemination whose primary tumor was classified as resectable/borderline resectable disease.

## Graphical abstract

The effectiveness of intraperitoneal chemotherapy using paclitaxel (i.p.‐PTX) in pancreatic ductal adenocarcinoma (PDAC) patients with peritoneal dissemination remains elusive. This study indicated that the i.p.‐PTX therapy may improve survival in selected patients with PDAC, in particular those with occult peritoneal dissemination.

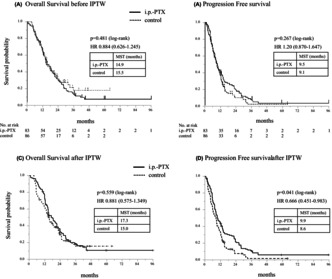

## 1 INTRODUCTION

Pancreatic ductal adenocarcinoma (PDAC) remains a highly lethal disease.^1^ Expected to become one of the leading causes of cancer‐related mortality by 2030, PDAC has a 5‐year overall survival (OS) of approximately 9%.^2^ Only 15%–20% of patients are eligible to undergo potentially curative oncologic resection, as most tumors are deemed unresectable at the time of diagnosis due to locally advanced disease or distant metastases.^3^

Gemcitabine has been the standard therapy for locally advanced or metastatic PDAC for the past two decades.^4^ The development of two first‐line combination chemotherapeutic regimens, FOLFIRINOX (oxaliplatin, irinotecan, fluorouracil and leucovorin) and gemcitabine plus nab‐paclitaxel (GnP), has significantly improved OS in patients with unresectable PDAC.^5^, ^6^ These regimens delay the deterioration of quality of life for patients with metastatic PDAC, improving the chance for salvage chemotherapy after progression on first‐line treatment as standard chemotherapy.^7^, ^8^, ^9^

Peritoneal dissemination is classified as macroscopic, appearing as peritoneal deposits, and microscopic, presenting as cancer cells in ascites or peritoneal lavage (CY+). In a population‐based study from the Netherlands, peritoneal dissemination was diagnosed in 7.7% of patients with PDAC, and their median survival time (MST) was only 3.4 months (pancreatic head tumor), 2.3 months (pancreatic body tumor), and 2.2 months (pancreatic tail tumor).^10^ An important reason for the limited effectiveness of systemic chemotherapy is the existence of the plasma‐peritoneal barrier, which limits the access of chemotherapy to cancer stem cells in the peritoneal cavity.^11^, ^12^ To overcome the plasma‐peritoneal barrier, various techniques for administering intraperitoneal liquid chemotherapy have been explored, and intraperitoneal chemotherapy has been reported to be more effective than systemic chemotherapy alone.^13^ Our previous Phase II multicenter trials evaluating intraperitoneal paclitaxel (i.p.‐PTX) combined with systemic chemotherapy showed acceptable toxicity and favorable activity against peritoneal dissemination in patients with PDAC.^14^, ^15^ Results of a retrospective study at a single institution demonstrated better symptom relief and survival benefits in patients who received i.p.‐PTX versus systemic chemotherapy before the introduction of FOLFIRINOX and GnP.^16^

In this retrospective, multicenter study, we compared the clinical effectiveness of i.p.‐PTX combined with systemic chemotherapy versus current standard systemic chemotherapy including FOLFIRINOX and GnP in patients with PDAC with occult peritoneal dissemination but without distant organ metastases.

## 2 METHODS

### 2.1 Patients

This was a retrospective analysis of data collected from 28 academic or community centers in Japan from November 2012 to December 2019 (the *Study Group of Pancreatic Ductal Adenocarcinoma with Peritoneal Dissemination in Japan*). All patients were diagnosed with PDAC using histological or cytological examination under staging laparoscopy or open laparotomy based on the following: (i) presence of microscopic peritoneal dissemination during staging laparoscopy in patients with radiographically defined, unresectable (UR)‐locally advanced (LA) PDAC at the primary tumor or (ii) presence of macroscopic peritoneal dissemination on staging laparoscopy or open laparotomy in patients with PDAC of any resectability status according to the National Comprehensive Cancer Network (NCCN) guidelines.^17^ Presence of other sites of distant metastases (excluding the ovaries), positive peritoneal cytology in patients without peritoneal deposits in otherwise resectable/borderline resectable (R/BR) PDAC, and active concomitant malignancies, were exclusion criteria. Eligible patients received i.p.‐PTX combined with systemic chemotherapy (i.p.‐PTX group) and systemic chemotherapy with FOLFIRINOX or GnP (control group). Administration of i.p. PTX was based on the previous studies,^14^, ^15^ and FOLFIRINOX or GnP was administered in a standard fashion. Enhanced abdominal CT and chest CT for assessing tumor response and tumor markers were carried out every 8 to 12 weeks in all patients. To evaluate the antitumor effects on peritoneal dissemination, peritoneal washing cytology through a peritoneal access port was examined using Papanicolaou and May‐Giemsa staining every 2 months, as previously reported.^14^, ^15^

### 2.2 Study endpoints

The primary endpoint was OS, which was defined as the time from staging laparoscopy or open laparotomy to diagnose peritoneal dissemination to death from any cause. Secondary endpoints were progression‐free survival (PFS), clinical response rate, adverse events (Common Terminology Criteria for Adverse Events, version 4.0.), and resection rate. Disease progression was defined as multiple organ metastasis such as liver, lung, peritoneal metastasis, or loco‐regional recurrence on contrast enhanced CT or MRI. Surgical resection was planned when patients with peritoneal dissemination were surgically fit and had declining tumor marker levels during chemotherapy, negative peritoneal washing cytology and disappearance of peritoneal deposits, and downstaging to potentially resectable disease. Objective tumor responses were classified according to the Response Evaluation Criteria in Solid Tumors (RECIST) guidelines, version 1.1.^18^ These endpoints were compared statistically between the i.p. PTX group and the control group.

### 2.3 Data collection

Case report forms contained 140 data fields per patient, including clinical background characteristics, perioperative data, pathological diagnosis, and survival period, and were designed to maximize accurate data collection with pop‐up boxes to define complications and grading systems. A total of 47 continuous variables, 73 categorical variables, and 20 free comments from the database were required for the statistical analyses conducted in this study.

### 2.4 Statistical analysis

For categorical variables, the chi‐square test was used to examine differences between groups; for numerical variables and nonparametric independent samples, the Mann–Whitney U test was used. Survival curves were calculated with the Kaplan–Meier method, and differences were compared with the log‐rank test. Hazard ratios (HR) with 95% confidence intervals (CI) and two‐sided *P*‐values were reported. HR in subgroups according to baseline characteristics and two‐tailed 95% CI were calculated with the Cox proportional hazards model. A *P*‐value of less than 0.05 was considered statistically significant. All statistical analyses were performed with JMP Pro version 14.0 (SAS Institute, Cary, NC, USA).

A propensity score approach was chosen over regular covariate adjustment to attenuate confounding by indication in the observational setting, while limiting the risks of overfitting and model misspecification.^19^ We used inverse probability of treatment weighting (IPTW) analysis instead of other propensity score methods (e.g., propensity score matching, stratification, or adjustment) because it is more appropriate for the estimation of the average treatment effect, which is the estimate that fit our objectives^20^ and seemed to perform best in our setting (i.e., survival outcomes with considerable overlap in propensity score).^21^ In IPTW analysis, the entire set of observed data is utilized for analysis. While other methods typically exclude data that are difficult to match to the treatment and control groups, IPTW adjusts for these by weighting. Since fewer samples are excluded, the statistical efficiency of the estimation can be improved. A biostatistician (S.T.) was responsible for the statistical analysis.

### 2.5 Ethics statement

This study was reviewed and approved (No. 2020131) by the institutional review board of Kansai Medical University, Japan, and complied with the Strengthening the Reporting of Observational Studies in Epidemiology (STROBE) guidelines.^22^ All procedures in this study were performed in accordance with the guidelines of the Declaration of Helsinki.

## 3 RESULTS

### 3.1 Patient baseline characteristics

**FIGURE 1:**
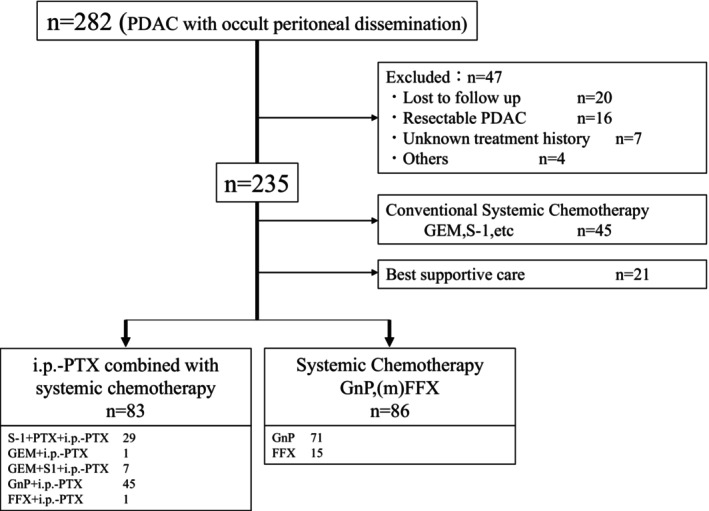
Flowchart of patient selection. PDAC, pancreatic ductal adenocarcinoma, GEM, gemcitabine, i.p., intraperitoneal, PTX, paclitaxel, GnP, gemcitabine plus nab‐paclitaxel, (m)FFX, modified FOLFIRINOX.

A total of 282 patients were eligible, and data from 169 patients were analyzed, as shown in the flowchart in Figure 1; all patients were diagnosed between November 2012 and December 2019. The median follow‐up duration was 18.2 months (range 7.8–95.6 months). The i.p.‐PTX group included 83 patients who received several regimens, including S‐1 plus PTX plus i.p.‐PTX (*n* = 29), gemcitabine plus i.p.‐PTX (*n* = 1), gemcitabine plus S‐1 plus i.p.‐PTX (*n* = 7), GnP plus i.p.‐PTX (*n* = 45), and FFX plus i.p.‐PTX (n = 1). The control group included 86 patients who received GnP (*n* = 71) or modified FOLFIRINOX (*n* = 15) as first‐line systemic chemotherapy. Patient characteristics are summarized in Table 1. Tumor size, CA19‐9 level, proportion of pancreas body and tail (Pbt), UR‐LA disease at the primary tumor, and presence of ascites were significantly higher in the i.p.‐PTX group than in the control group (*p* < 0.05).

**TABLE 1:** Patient characteristics.

Pre‐treatment factors	i.p.‐PTX (*n* = 83)	Control (*n* = 86)	*p*‐value
Sex, Male: Female, *n* (%)	40 (48): 43 (52)	52 (60): 34 (40)	0.109
Age, median (range), years	67 (42–82)	69 (37–82)	0.440
Performance status, 0:1, *n* (%)	68 (82): 15 (18)	72 (84): 14 (16)	0.757
Modified Glasgow prognostic score, 0:1:2, *n* (%)	70 (81): 3 (4): 13 (15)	65 (78): 9 (11): 9 (11)	0.135
Prognostic Nutritional Index, median (range)	43.9 (28.5–59.0)	44.3 (29.9–55.8)	0.714
Primary tumor site, Ph: Pbt, *n* (%)	21 (25): 62 (75)	40 (46): 46 (54)	0.004
Radiological tumor size, median (range), mm	40 (8–105)	32 (12–85)	0.022
NCCN resectability status of primary tumor, R/BR: UR‐LA, *n* (%)	34 (41): 49 (59)	50 (58); 36 (42)	0.025
Peritoneal nodule, *n* (%)	62 (75)	67 (78)	0.623
Peritoneal nodule. 0: 1: 2–5: >6, *n* (%)	21 (25): 7 (8): 13 (16): 42 (51)	19 (22): 11 (13): 22 (26): 34 (40)	0.248
Ascites, *n* (%)	42 (51)	28 (33)	0.017
CA19‐9, median (range), U/mL	598 (1–38 000)	175 (0.6–30 197)	0.005

Abbreviations: BR, borderline resectable; NCCN, National Comprehensive Cancer Network; Pbt, pancreas body and tail; Ph, pancreas head; R, resectable; UR‐LA, unresectable locally advanced tumor.

### 3.2 Post‐treatment outcomes

As shown in Table 2, the −16.7% (range − 81% to 75%) rate of radiological tumor shrinkage in the i.p.‐PTX group was significantly greater compared with that in the control group, −8.0% (range − 69% to 29%; *p* = 0.034). A nearly 60% decrease in CA19‐9 level was found in both groups, showing no significant difference between groups. Conversion surgery was performed in 19% (16 of 83) of patients in the i.p.‐PTX group, which was significantly more common than the 5% (four of 86) of patients in the control group (*p* = 0.003). Peritoneal washing cytology became negative in 55% of patients in the i.p.‐PTX group at a median of 4.0 months (range 0.9–26.8) after introduction of i.p.‐PTX. There were no significant differences between groups in the location of cancer progression, frequency of second‐line chemotherapy, requirement for abdominal paracentesis, and development of intestinal obstruction.

**TABLE 2:** Post‐treatment outcomes.

Post‐treatment factor	i.p.‐PTX (*n* = 83)	Control (*n* = 86)	*p*‐value
1st‐line chemotherapy regimen, *n*			
S‐1 + PTX + PTXip	29	/	
GEM+PTXip	1	/	
GEM+S‐1 + PTXip	7	/	
GnP + PTXip	45	/	
FFX + PTXip	1	/	
GnP	/	71	
FFX	/	15	
Radiological tumor size (best response), median (range), mm	27 (8–81)	28 (10–81)	0.537
Radiological tumor shrinkage rate, median (range), %	−16.7% (−81% to 75%)	−8.0% (−69% to 29%)	0.034
Best response RECIST, CR/PR: SD/PD, *n* (%)	29 (35): 54 (65)	20 (23): 66 (77)	0.094
CA19‐9 response from baseline, median (range), %	−57.6% (−99.9% to 259%)	−60.9% (−98.9% to 183%)	0.324
Peritoneal cytology becoming negative, *n* (%)	44 (55%) (*n* = 80)	/	
Time of peritoneal cytology becoming negative, median (range), months	4.0 (0.9–26.8)	/	
Conversion surgery, *n* (%)	16 (19)	4 (5)	0.003
Location of cancer progression			
Liver metastasis, *n* (%)	7 (8)	7 (8)	0.945
Primary tumor growth, *n* (%)	23 (28)	26 (30)	0.718
Exacerbation of peritoneal dissemination, *n* (%)	14 (17)	19 (22)	0.391
Elevation of CA19‐9, *n* (%)	18 (27)	12 (14)	0.187
2nd‐line treatment, *n* (%)	43 (64)	49 (60)	0.580
2nd‐line chemotherapy regimen, *n*			
GnP	14	9	
mFFX	17	15	
GEM	1	4	
S‐1	6	14	
GEM+S‐1	2	1	
GEM+erlotinib	1	1	
Others	2	5	
Abdominal paracentesis, *n* (%)	14 (17)	14 (16)	0.918
Intestinal obstruction, *n* (%)	14 (17)	16 (19)	0.768
Time of emergence of intestinal obstruction, median (range), months	11.9 (0–31.0)	14.3 (5.3–28.3)	0.662

Abbreviations: CR, complete response; GEM, gemcitabine; GnP, gemcitabine plus nab‐paclitaxel; mFFX, modified FOLFIRINOX; PD, progressive disease; PR, partial response; PTX, paclitaxel; PTXip, intraperitoneal chemotherapy using paclitaxel; SD, stable disease.

### 3.3 Adverse events

As shown in Table SS1, the most common grade 3 or 4 hematologic toxicities in both the i. p‐PTX and control groups were leukopenia and neutropenia. Grade 3 or 4 leukopenia (54% vs. 30%, respectively; *p* = 0.005) and neutropenia (59% vs. 40%, respectively; *p* = 0.024) developed more frequently in the i. p‐PTX group than in the control group. Peripheral neuropathy was less frequently found in the i. p‐PTX group than in the control group (5% vs. 29%, respectively; *p* < 0.001). The incidences of the other adverse events assessed were similar between groups.

### 3.4 Endpoint analysis

**FIGURE 2:**
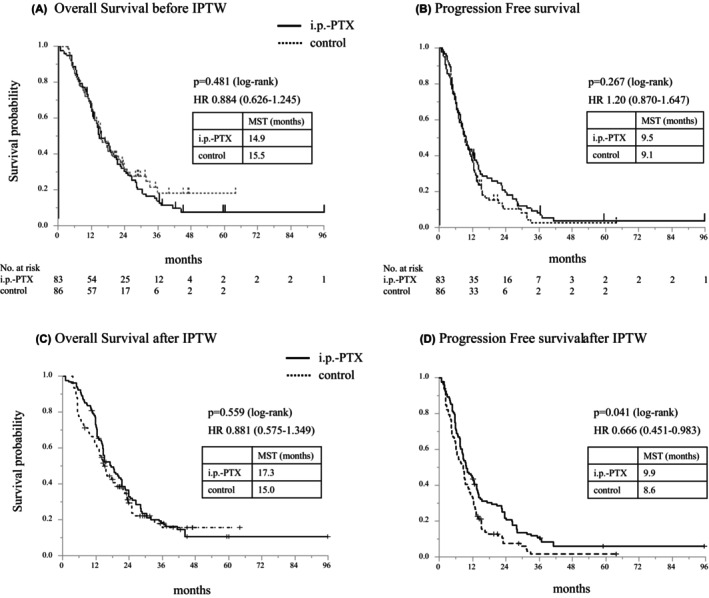
Survival analysis before and after IPTW. Kaplan–Meier curves show (A) overall survival before IPTW, (B) progression‐free survival before IPTW, (C) overall survival after IPTW, and (D) progression‐free survival after IPTW. HR, hazard ratio, IPTW, inverse probability of treatment weighting, i.p.‐PTX, intraperitoneal paclitaxel, MST, median survival time.

Patients receiving intraperitoneal chemotherapy had similar median OS compared with those receiving standard systemic chemotherapy in the total cohort (i.p.‐PTX, 14.9 months [95% CI: 12.2–20.1] vs. control, 15.5 months [95% CI: 13.0–19.3, HR: 0.884 (0.626–1.245)] *p* = 0.481, Figure 2A). In the control group, MST in the 71 patients who received GnP was similar to that in the 15 patients who received modified FOLFIRINOX (15.4 v.s. 15.5 months, respectively; *p* = 0.467). The median PFS was 9.5 months (95% CI, 7.6 to 12.3) in the i. p‐PTX group and 9.1 months (95% CI, 7.0 to 11.4) in the control group (HR:1.200 [0.870–1.647] *p* = 0.267, Figure 2B) in the total cohort.

When patient characteristics were adjusted with the IPTW approach, all variables were well balanced in the weighted cohort as standardized differences decreased (Table S2). The median OS of the i.p.‐PTX and control groups was not statistically different (17.3 months [95% CI, 14.3 to 23.6] vs. 15.0 months [95% CI, 11.2 to 23.1], respectively; *p* = 0.559, Figure 2C). The PFS in the i.p.‐PTX group was significantly longer than that in the control group (median 9.9 months [95% CI, 7.8 to 14.0] vs. 8.6 months [95% CI, 5.9 to 11.4], respectively; *p* = 0.041, Figure 2D).

**FIGURE 3:**
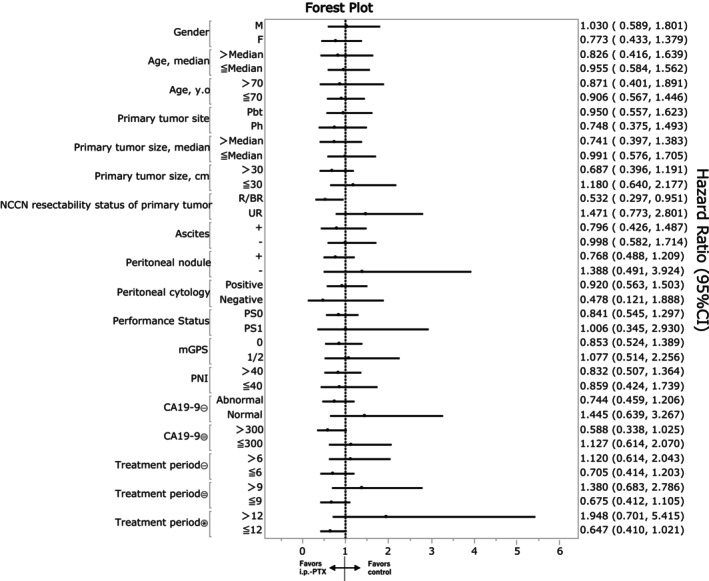
Forest plot of overall survival in subgroups of the entire cohort. CI, confidence interval, i.p.‐PTX, intraperitoneal paclitaxel, mGPS, modified Glasgow prognostic score, NCCN, National Comprehensive Cancer Network, Pbt; pancreas body and tail, Ph, pancreas head, PNI, Prognostic Nutritional Index, PS, performance status, R/BR, resectable/borderline resectable, UR, unresectable. Treatment period means duration from the date starting first‐line chemotherapy to the date of the final administration of the anticancer drug.

**FIGURE 4:**
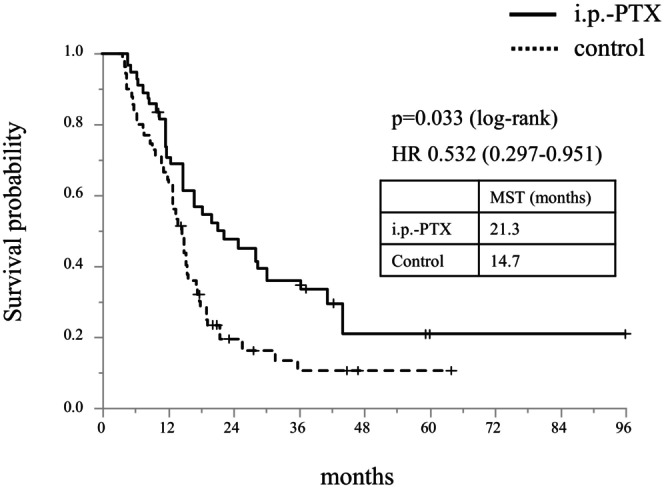
Survival analysis of patients with resectable/borderline resectable disease after IPTW. HR, hazard ratio, IPTW, inverse probability of treatment weighting, i.p.‐PTX, intraperitoneal paclitaxel, MST, median survival time, R/BR, resectable/borderline resectable.

Subgroup analysis indicated that the OS between the i.p.‐PTX and control groups was also comparable in most subgroups, except for patients with R/BR disease at the primary tumor (Figure 3). Among them, matched patients in the i. p‐PTX group had significantly longer MST than those in the control group (21.3 vs. 14.7 months, respectively; *p* = 0.033, Figure 4). Table 3 shows significantly higher proportions of pancreatic body and tail cancer, presence of ascites, and number of peritoneal nodules in the i.p.‐PTX group than in the control group (*p* < 0.05). Although advanced‐stage peritoneal dissemination was frequently found in the i.p.‐PTX group, conversion surgery was performed in the i.p.‐PTX group significantly more often than in the control group (24% vs. 4%, respectively; *p* = 0.006).

**TABLE 3:** Characteristics of patients with R/BR disease.

Pre‐treatment factors	i.p.‐PTX (*n* = 34)	Control (*n* = 50)	*p*‐value
Sex, Male: Female, *n* (%)	14 (41): 20 (59)	29 (58): 21 (42)	0.129
Age, median (range), years	66 (46–8)	70 (37–80)	0.208
Performance status, 0:1, *n* (%)	26 (76): 8 (24)	43 (86): 7 (14)	0.267
Modified Glasgow prognostic score, 0:1:2, *n* (%)	30 (88): 0 (0): 4 (12)	43 (86): 1 (2): 6 (12)	0.592
Prognostic Nutritional Index, median (range)	44.2 (34.0–59.0)	44.2 (31.7–55.8)	0.572
Primary tumor site, Ph: Pbt, *n* (%)	6 (18): 28 (82)	23 (46): 27 (54)	0.006
Radiological tumor size, median (range), mm	36 (8–74)	30 (12–77)	0.061
Peritoneal nodule. 1: 2–5: >6, *n* (%)	0 (0): 7 (21): 27 (79)	9 (18): 14 (28): 27 (54)	0.003
Ascites, *n* (%)	20 (59)	13 (26)	0.002
Pretreatment (within 3 months), *n* (%)	9 (26)	10 (20)	0.601
CA19‐9, median (range), U/mL	623 (1–19 530)	210 (0.6–29 582)	0.068
Radiological tumor size (best response), median (range), mm	27 (8–59)	27 (10–81)	0.465
Radiological tumor shrinkage rate, median (range), %	−10.0% (−81% to 75%)	−5.9% (−58% to 29%)	0.202
Best response RECIST, CR/PR: SD/PD, *n* (%)	9 (26): 25 (74)	12 (24): 38 (76)	0.952
CA19‐9 response from baseline, median (range), %	−55.1% (−97.6% to 259%)	−49.7% (−98.9% to 167%)	0.890
Peritoneal cytology becoming negative, *n* (%)	20 (61)	/	
Time of peritoneal cytology becoming negative, median (range), months	3.9 (1.9–7.6)	/	
Conversion surgery, *n* (%)	8 (24)	2 (4)	0.006

Abbreviations: CR, complete response; i.p.‐PTX, intraperitoneal paclitaxel; Pbt, pancreas body and tail; PD, progressive disease; Ph, pancreas head; PR, partial response; SD, stable disease.

### 3.5 Conversion surgery

**FIGURE 5:**
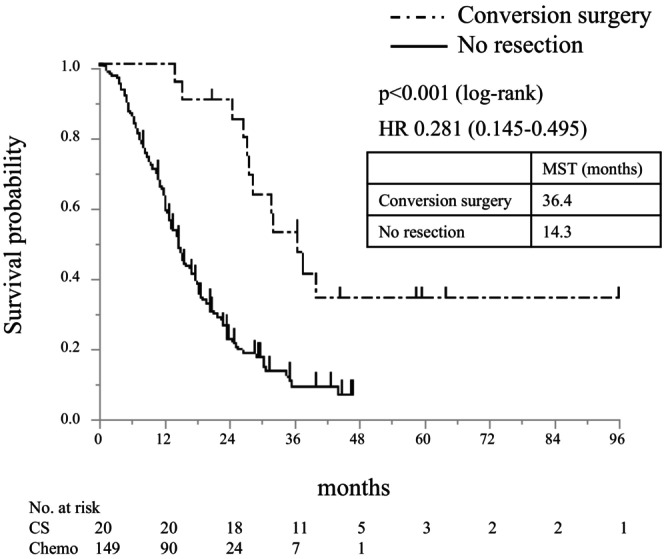
Survival analysis by surgery status. Overall survival from initial treatment. Chemo, chemotherapy, CS, conversion surgery, HR, hazard ratio, MST, median survival time.

Table S3 summarizes the clinical data of 20 patients who underwent conversion surgery (16 patients in the i.p.‐PTX group and four patients in the control group) after confirming macroscopic and microscopic disappearance of peritoneal dissemination, declined tumor marker levels, and stable performance status. The median time to resection was 9.5 months (range 4.1–15.1 months). Six patients underwent pancreaticoduodenectomy, 10 received distal pancreatectomy, three received distal pancreatectomy with celiac axis resection, and one received total pancreatectomy. Eighteen patients (80%) achieved R0 resection, and 13 patients (65%) had no lymph node metastasis. There was no postoperative mortality, and 18 patients received adjuvant chemotherapy (intraperitoneal chemotherapy in 12 patients and standard systemic chemotherapy in six patients). As shown in Figure 5, the MST from initial treatment in patients who underwent conversion surgery was 36.4 months (95% CI: 27.9–not calculated), which was significantly better than that of patients who did not undergo conversion surgery (14.3 months [12.2–15.7], *p* < 0.001). The MST from conversion surgery was 25.0 months (95% CI: 17.6–not calculated).

## 4 DISCUSSION

The peritoneum is the second most common metastatic site following the liver in PDAC, and the development of peritoneal dissemination significantly contributes to the devastating prognosis. In a population‐based study from the Netherlands, the MST of patients with PDAC with peritoneal metastasis was only 2.2 to 3.4 months.^10^ Systemic chemotherapy is less effective against peritoneal dissemination than hematogenous metastases. The efficacy of anticancer drugs generally depends on their concentration and duration of tumor exposure. However, peritoneal penetration of conventional chemotherapy is poor, and only a small fraction of the systemically administered drug is delivered to the peritoneum. Intraperitoneal administration of anticancer drugs enables an extremely high concentration of drugs to contact the target cancer lesions directly in the peritoneal cavity.^23^ Some studies, including phase II trials of intraperitoneal PTX combined with systemic chemotherapy, showed promising results in terms of response rate and survival time in patients with malignant ascites or occult peritoneal dissemination.^14^, ^15^, ^16^, ^24^, ^25^ However, there is no comparative study between i.p.‐PTX and recent chemotherapy regimens, such as GnP or FOLFIRINOX, in patients with peritoneal dissemination. Therefore, we conducted this retrospective multicenter study to evaluate the clinical effectiveness of i.p.‐PTX. Recruited patients were limited to those with occult peritoneal dissemination diagnosed under staging laparoscopy, bypass surgery, or open laparotomy, but not with imaging studies. As a result, the OS and PFS curves in the i.p.‐PTX group were nearly identical to those in the control group in the unadjusted analysis, showing worse tumor staging in the i.p.‐PTX group. Differences in clinical background and first‐line systemic chemotherapy might affect OS and PFS. Tumor size, CA19‐9 level, and the proportion of Pbt, UR‐LA at the primary tumor and presence of ascites were significantly higher in the i.p.‐PTX group than in the control group. Although Grade 3 or 4 leukopenia and neutropenia developed more frequently in the i.p.‐PTX group than in the control group, peripheral neuropathy was less common in the i.p.‐PTX group than in the control group. Adverse events can be more easily controlled in i.p.‐PTX regimens than in GnP or FOLFIRINOX regimens because peripheral neuropathy persists for a long time, resulting in patient discomfort and decreased quality of life.

The present study contained various biases because of its retrospective design. We used the IPTW method to make weighted cohorts in which patient characteristics had the same distribution. We minimized the effects of confounding factors as much as possible and obtained well‐balanced estimates of average treatment effects. After adjusting for clinical background, the IPTW approach showed longer PFS, but not OS, in the i.p.‐PTX group. Conversion surgery with high rates of radiological tumor shrinkage was performed more often in the i.p.‐PTX group (19%) than in the control group (5%). Treatment with i.p.‐PTX may control the progression of peritoneal dissemination in addition to that of the primary tumor. However, no significant difference in OS was found between groups, even after IPTW. OS in the i.p.‐PTX group (MST, 14.9 months) was similar to that reported previously (MST, 14.5–17.9 months).^14^, ^15^, ^16^ OS in the control group appeared much better in this study with FFX and GnP (MST, 15 months) than in previous reports (MST, 10 months). Despite the i.p. PTX group has advantage in a use of FOLFIRINOX or GnP regimen as second‐line chemotherapy, FOLFIRINOX and GnP as first‐line or second‐line chemotherapy in the control group could control even peritoneal dissemination. The reason why OS extension was not observed despite PFS extension in the i.p. PTX group might be explained favorable effects of FOLFIRINOX or GnP regimen as the first‐ or second‐line chemotherapy on OS in the control group, and high proportion of conversion surgery resulting in prolonged PFS in the i.p. PTX group. However, OS in patients with R/BR disease at the primary tumor was significantly better in the i.p.‐PTX group than in the control group, as shown in the forest plot analysis. These findings in the i.p.‐PTX group may be associated with the high incidence of conversion surgery, which is associated with better OS. Conversion surgery was performed in 24% of patients with R/BR disease at the primary tumor, which was more often than in 16% of patients with LA disease at the primary tumor in the i.p.‐PTX group. In contrast, in the control group, the frequency of conversion surgery was 4% (two patients) in patients with R/BR disease at the primary tumor, which was similar to the 6% (two patients) in patients with LA disease at the primary tumor in the control group. In this setting, the i.p.‐PTX regimen may enhance control of peritoneal dissemination.

Surgical resection is not usually performed for patients with metastatic PDAC. According to international guidelines for the treatment of PDAC and widespread clinical practice, resection of pancreatic cancer metastases is not recommended and therefore not routinely performed in clinical practice.^26^, ^27^ However, recent developments in chemotherapy may provide more opportunities for potentially curative resection, even in carefully selected patients with metastatic PDAC.^28^, ^29^, ^30^, ^31^ The evidence for selection of patients eligible for this aggressive approach is based primarily on small case series.^29^, ^30^ In the current study, effective elimination of peritoneal deposits and intraperitoneal free cancer cells allowed us to perform conversion surgery in selected patients. MST from initial treatment in patients who underwent conversion surgery was 36.4 months, which was significantly longer than that of patients who did not undergo conversion surgery (MST, 14.3 months [range 12.2–15.7 months], *p* < 0.001). Yamada et al. reported that conversion surgery after intraperitoneal treatment resulted in promising clinical effectiveness with acceptable tolerability in patients with PDAC with peritoneal dissemination.^32^ As stated in the Japanese clinical practice guideline, conversion surgery should be performed in patients whose peritoneal dissemination becomes undetectable macroscopically and microscopically.^33^ Treatment with i.p.‐PTX may increase the potential of conversion surgery to prolong survival in patients with PDAC with peritoneal dissemination, especially those with R/BR PDAC at the primary tumor.

The strength of this study is that it is the first report showing clinical outcomes in patients with PDAC with occult peritoneal dissemination with an i.p.‐PTX regimen and systemic chemotherapy with modern regimens based on data from multiple institutions in Japan. There are several confounding issues in interpreting these results. One limitation is the nonrandomized design. Major selection bias was caused by the different enrollment periods. There may be potential bias caused by differences in drug dosage or duration of administration between two groups. Various clinicopathological factors relevant to decision making, especially for conversion surgery, were not controlled as strictly as in a randomized clinical trial. In the control group, the peritoneal port was not routinely placed, and therefore, number of patients with negative peritoneal cytology might be underestimated, resulting in the lower proportion of conversion surgery in the control group. Despite efforts to control for baseline factors with IPTW, this was not a prospective randomized trial, and the two groups of patients were not the same. Furthermore, the characteristics of the enrolled patients may have been influenced by referral bias, because this was a multi‐institutional study.

In conclusion, this study did not demonstrate a survival benefit of ip‐PTX compared to GnP or FFX in terms of OS. However, IPTW analysis revealed a prolongation effect on PFS, and subgroup analysis suggested the possibility of an OS benefit in patients with occult peritoneal dissemination whose primary tumor was classified as R/BR. Currently, we are conducting a phase III randomized controlled trial to compare OS between S‐1 plus intravenous/i.p.‐PTX and GnP in patients with PDAC with occult peritoneal dissemination (UMIN000027229/jRCTs051180199).^34^

## AUTHOR CONTRIBUTIONS

**Tomohisa Yamamoto:** Conceptualization; data curation; formal analysis; investigation; methodology; project administration; writing – original draft. **Toshio Shimokawa:** Conceptualization; formal analysis; investigation; methodology; project administration; software; supervision; validation; writing – review and editing. **Masamichi Hayashi:** Data curation; formal analysis; investigation; project administration; supervision; visualization; writing – review and editing. **Masamichi Mizuma:** Conceptualization; data curation; formal analysis; investigation; project administration; supervision; visualization; writing – review and editing. **Katsuhisa Hirano:** Data curation; formal analysis; investigation; project administration; writing – review and editing. **Atsushi Oba:** Data curation; investigation; resources; supervision; visualization; writing – review and editing. **Toshimichi Asano:** Data curation; investigation; methodology; project administration; writing – review and editing. **Hideyo Miyato:** Data curation; investigation; project administration; writing – review and editing. **Makoto Yoshida:** Data curation; investigation; project administration; writing – review and editing. **Ippei Matsumoto:** Data curation; investigation; methodology; project administration; resources; supervision; writing – review and editing. **Yasunari Kawabata:** Data curation; investigation; visualization; writing – review and editing. **Katsunori Sakamoto:** Data curation; investigation; writing – review and editing. **Fuyuhiko Motoi:** Conceptualization; formal analysis; investigation; methodology; project administration; resources; supervision; visualization; writing – review and editing. **Shigeto Ishii:** Data curation; investigation; resources; writing – review and editing. **Yuki Homma:** Data curation; investigation; project administration; writing – review and editing. **Hiromitsu Maehira:** Data curation; investigation; resources; writing – review and editing. **Yutaro Matsunaga:** Data curation; investigation; resources; writing – review and editing. **Tetsuya Ikemoto:** Data curation; investigation; writing – review and editing. **Masafumi Nakamura:** Data curation; investigation; methodology; project administration; resources; writing – review and editing. **Yuko Mataki:** Data curation; investigation; resources; writing – review and editing. **Tsuyoshi Notake:** Data curation; investigation; writing – review and editing. **Keiichi Akahoshi:** Data curation; investigation; methodology; resources; writing – review and editing. **Hideki Takami:** Data curation; investigation; resources; writing – review and editing. **So Yamaki:** Data curation; investigation; writing – review and editing. **Daisuke Hashimoto:** Data curation; investigation; writing – review and editing. **Yasutoshi Kimura:** Data curation; investigation; methodology; resources; writing – review and editing. **Satoshi Hirano:** Conceptualization; investigation; methodology; project administration; resources; supervision; writing – review and editing. **Yosuke Inoue:** Data curation; investigation; methodology; resources; supervision; visualization; writing – review and editing. **Tsutomu Fujii:** Conceptualization; formal analysis; investigation; methodology; project administration; resources; supervision; validation; visualization; writing – review and editing. **Michiaki Unno:** Conceptualization; investigation; methodology; resources; supervision; writing – review and editing. **Yasuhiro Kodera:** Conceptualization; investigation; methodology; project administration; resources; supervision; visualization; writing – review and editing. **Joji Kitayama:** Conceptualization; data curation; investigation; methodology; project administration; resources; supervision; validation; visualization; writing – review and editing. **Sohei Satoi:** Conceptualization; data curation; formal analysis; funding acquisition; investigation; methodology; project administration; resources; supervision; validation; visualization; writing – original draft; writing – review and editing.

## FUNDING INFORMATION

The authors did not receive support from any organization for the submitted work.

## CONFLICT OF INTEREST STATEMENT

Satoi S discloses research funding from Nihon Servier and Amino‐up Co. Motoi F received honoraria and scholarship from Taiho pharma co. Nakamura M disclose research funding from Taiho pharma co. Unno M disclose research funding from Taiho pharma co. Fujii T serves as an associate editor for *Annals of Gastroenterological Surgery*. Kodera Y received payment or honoraria from Taiho pharma co, Eli Lilly Japan K.K., and Pfizer Japan Inc., and serves as an associate editor for *Annals of Gastroenterological Surgery*.

## Supporting information


Table S1.
Table S2.
Table S3.
