# Supplementary material for: Clinical role of intraperitoneal chemotherapy in patients with pancreatic ductal adenocarcinoma concomitant with occult peritoneal dissemination: A multicenter retrospective study
Source: Ann Gastroenterol Surg. 2025 Mar 4;9(4):830–41. doi: 10.1002/ags3.70001 (PMC12211105; doi:10.1002/ags3.70001)
Supplement: Supplementary file 1 — Table S1. Table S2. Table S3. [file AGS3-9-830-s001.docx]

**Supplemental table 1 Adverse events**

|  | **i.p.-PTX (n=83)** | **Control (n=86)** | ***P*-value** |
| --- | --- | --- | --- |
| ***Hematologic toxicity (≥Grade3)*** |  |  |  |
| Leucocytopenia | 44 (54) | 26 (30) | 0.005 |
| Neutropenia | 49 (59) | 34 (40) | 0.024 |
| Febrile neutropenia | 9 (11) | 6 (7) | 0.350 |
| Anemia | 6 (7) | 12 (14) | 0.176 |
| Thrombocytopenia | 4 (5) | 4 (5) | 0.507 |
| ***Nonhematologic toxicity (≥Grade2)*** |  |  |  |
| Appetite loss | 12 (14) | 18 (21) | 0.252 |
| Vomiting | 4 (5) | 6 (7) | 0.412 |
| Diarrhea | 8 (10) | 6 (7) | 0.425 |
| Skin rash | 2 (2) | 1 (1) | 0.422 |
| Mucositis oral | 2 (2) | 0 (0) | 0.123 |
| Peripheral neuropathy | 4 (5) | 25 (29) | <0.001 |
| Alopecia | 20 (24) | 29 (34) | 0.168 |
| Pneumonia | 3 (4) | 6 (7) | 0.317 |
| Dysgeusia | 1 (1) | 4 (5) | 0.191 |

**Supplemental table 2 Baseline characteristics after inverse probability treatment weighting (IPTW)**

|  | **Weighted data** | | | |
| --- | --- | --- | --- | --- |
|  | **i.p.-PTX**  **(n=83)** | **control**  **(n=84)** | ***P*-value** | **Standardized difference.** |
| Sex, Male, n (%) | 48.6 (59.2) | 45.8 (52.7) | 0.493 | 0.131 |
| Age, years | 66.2 (8.5) | 66.7 (9.6) | 0.754 | 0.057 |
| Primary tumor site, Pbt, n (%) | 52.2 (63.6) | 55.8 (64.2) | 0.945 | 0.013 |
| Primary tumor size, mm (SD) | 38.3 (15.8) | 38.7 (16.5) | 0.914 | 0.024 |
| Resectability status of primary tumor, R/BR, n (%) | 38.9 (47.4) | 39.8 (45.8) | 0.866 | 0.032 |
| Ascites, n (%) | 33.6 (41.0) | 37.8 (43.5) | 0.790 | 0.052 |
| Peritoneal nodule, n (%) | 60.8 (74.0) | 60.6 (69.8) | 0.658 | 0.095 |
| PS=1, n (%) | 13.0 (15.9) | 18.4 (21.2) | 0.543 | 0.137 |
| mGPS=0, value (SD) | 67.2 (81.8) | 72.9 (83.9) | 0.740 | 0.055 |
| PNI, value (SD) | 43.4 (6.0) | 43.0 (8.7) | 0.757 | 0.050 |
| Log CA19-9, value (SD) | 5.6 (2.2) | 5.6 (2.8) | 0.973 | 0.007 |

Pbt, pancreas body and tail; R, resectable; BR, borderline resectable; PS, Performance status; mGPS, modified glasgow prognostic score; PNI, Prognostic Nutritional Index; SD, standard deviation

**Supplemental table 3 Clinical outcomes of patients who underwent conversion surgery**

| ***Parameter*** | **n=20** |
| --- | --- |
| Time from initial treatment to conversion surgery, median (range), months | 9.5 (4.1-15.1) |
| Surgical procedure, PD: DP: DP-CAR: TP, n (%) | 6 (30) :10 (50) :3 (15):1 (5) |
| Portal vein resection, n (%) | 8 (40) |
| Artery resection, n (%) | 3 (15) |
| Operation time, median (range), min | 346 (218-866) |
| Extent of blood loss median (range), mL | 671 (295-6301) |
| Pathological tumor size, median (range), mm | 26 (1-80) |
| pT (T1a, T1b, T1c, T2, T3), n (%) | 1 (5): 0 (0): 2 (10): 1 (5): 16 (80) |
| pN (N0: N1a: N1b), n (%) | 13 (65): 4 (20): 3 (15) |
| Evans grade, 1: 2a: 2b: 3: 4, n | 3 (15): 7 (35): 6 (3): 4 (20) |
| Residual tumor, R0: R1, n | 18 (90): 2 (10) |
| Post-operative complication（Clavien-Dindo）, 0-2: 3a: 3b: 4: 5, n | 17: 3: 0: 0: 0 |
| Adjuvant therapy, n (%) | 18 (90) |
| Adjuvant therapy using intraperitoneal treatment, n (%) | 12 (60) |

PD, pancreaticoduodenectomy; DP, distal pancreatectomy; DP-CAR, distal pancreatectomy with en-bloc celiac axis resection; TP, total pancreatectomy.
